# Editorial: Social media and political participation: unpacking the role of social media in contemporary politics

**DOI:** 10.3389/fsoc.2025.1658996

**Published:** 2025-08-01

**Authors:** Pradeep Nair, Tobias Eberwein, Cassian Scruggs Sparkes-Vian

**Affiliations:** ^1^Department of New Media, Central University of Himachal Pradesh, Dharamshala, India; ^2^Institute for Comparative Media & Communication Studies, Austrian Academy of Sciences, Vienna, Austria; ^3^ACE - Film and Journalism, University of the West of England, Bristol, United Kingdom

**Keywords:** social media, political participation, democratic discourse, citizen engagement, public sphere, political knowledge

The emergence of social media has altered how people will access and use news and information for participation and engagement in political discourse. The most preferred media platforms like Facebook, Twitter, Whatsapp and Instagram has not only allowed people to participate in real-time discussions and debates but have also given them an edge to share and consume political content across time and geographical boundaries (Ahmed, 2023). It further empowered the marginalized communities by allowing them to speak their opinions and interact with others who share their perspective without any compulsions (Borah et al., 2022).

Despite the increasing role of social media in active political governance and policy domain, its socio-political consequences remain less explored. There is no doubt that in recent times, social media has become a crucial force in contemporary political world with more insights on how people access and engage with political information in their day-to-day life (Hopp et al., 2023). A large number of people worldwide have their dependency on social media for access to news and information. This overwhelming response to social media as the most preferred platform for communication has spurred considerable scholarly interest in defining the role of social media in modern democracy (Hutchens et al., 2023).

This Research Topic collection provides a body of research having new insights on how social media proves itself as an effective tool for accomplishing political goals by raising public awareness, changing people's minds about issues, getting elected officials to pay attention to issues influencing policy decisions (Kim and Lee, 2021). The published Research Topic collection has studies having a diverse and vast coverage. The research article “*Informing vs. promoting. The use of TikTok on France TV, BBC, and SVT*” by Maroto-González et al. intends to understand the use of TikTok by public broadcasters—France TV, BBC, and SVT to measure its informational and promotional relevance on the platform, and to identify the types of videos that generate the highest levels of engagement among users. The study examines the news profile associated with the information and the corporate profile of each of the selected broadcasters. The findings of the study revealed an interest in both promotional and informational content shared by the platform on the TikTok. In the case of SVT, higher levels of engagement were observed in comparison to BBC and France TV profiles.

Another research titled “*The influence of multimodal connectedness on political participation in China: an empirical study of the O-S-R-O-R model based on the life span perspective*” by Li and Li talks about promoting the construction of internet democratic politics in China and how it requires an understanding of multimodal connectedness to enhance citizens' political participation. The study introduced an Orientation-Stimuli-Reasoning-Orientations-Responses (O-S-R-O-R) model explaining the pathway from multimodal connectedness to political participation. The study “*Exposure to diverse political views in contemporary media environments*” by Steinfeld and Lev-on explores the nexus between digital media and citizens' exposure to diverse political views. It urges for democratic engagement through digital media platforms. Contrary to the theories of echo chambers and filter bubbles, the study reveals a nuanced media landscape where digital platforms facilitate both homogeneous and heterogeneous political exposures. The research study by Elareshi et al. on “*Public engagement through public service advertisements for health care awareness during early COVID-19 in Pakistan*” talks about public engagement through public service advertisements and the effectiveness of health care awareness during early COVID-19 in Pakistan. The study finds that Public Service Advertisements (PSAs) have a noted impact on public perception about pandemic preparedness and they played an important role in spreading awareness in Pakistan. The study titled “*Role of public relations practices in content management: the mediating role of new media platforms*” by Al Hadeed et al. talks about the inter-linkages of media content management, audience and communication by explaining the role of public relations in media organizations of UAE. The study by Zimmermann et al. on “*Political news on Instagram: influencer versus traditional magazine and the role of their expertise in consumers' credibility perceptions and news engagement*” provides new insights on the increasing role of source expertise in terms of credibility perceptions and news engagement intentions especially in the context of Instagram. The study titled “*Savvy and woke: gender, digital profile, social media competence, and political participation in gender issues among young Filipino netizens*” by Dayrit et al. talks about exploring and determining the predictive relationship of gender, digital profile, and social media competence and their role in political participation among young Filipino netizens.

The research study “*Storm the Capitol: linking offline political speech and online Twitter extra-representational participation on QAnon and the January 6 insurrection*” by Lee et al. explores the link between offline political speech and online extra-representational participation by examining Twitter within the context of January 6 insurrection and finds that there is an urgent need of policy implications for the role of online messaging as a tool of political mobilization. The study “*Trust and engagement on Twitter during the management of COVID-19 Pandemic: the effect of gender and position*” by Yousefinaghani et al. examined the different socio-political contexts provided by Social Media in terms of gender and their positional role and concludes that Twitter has provided different gender roles to individuals to communicate and manage the pandemic preparedness. The last study of the Research Topic collection titled “*#funnypoliticians: how do political figures use humour on Twitter?*” by Mendiburo-Seguel et al. explores how politicians use aggressive humor in political tweets to attract audiences on Twitter and how it affects the audience engagement in political discourse.

We hope that this Research Topic collection will prove its worth for the readers by giving them some new insights on the opportunities and challenges of social media communication in political discourse. In this age of digital communication, we must acknowledge that the social media has proved its relevance in engaging a large number of people in political discourse by allowing marginalized voices to be heard. Due to its hyper-interactive nature, social media has the potential to draw attention of people to a large number of issues which were less reported and represented in mainstream commercial media (Theocharis et al., 2022). The variety and inclusiveness of political discourse on social media has given a larger representation of diverse view-points to be heard and contested. But at the same time, social media has also posed new challenges to political dialogues in terms of conspiracy theories, fake news, propagandas (Lee et al., 2024). The correspondence of users' pre-existing ideas with social media algorithms results in frequent emphasis of issues resulting in echo-chambers further dividing and polarizing diverse dialogues and views (Li, 2025). Despite these challenges, social media is till date the most preferred platform for political communication, participation and discourse, giving everyone a space and sphere to communicate their viewpoints across geographies, ideologies and spaces with a sense of engagement and ownership.
